# Analysis of genes for alcoholism using two-disease-locus models

**DOI:** 10.1186/1471-2156-6-S1-S149

**Published:** 2005-12-30

**Authors:** Chih-Chieh Wu, Sanjay Shete

**Affiliations:** 1 Department of Epidemiology, The University of Texas M.D. Anderson Cancer Center, Houston, Texas, USA

## Abstract

Using model-based two-locus methods for mapping genes, we analyzed the family data from the Collaborative Study on the Genetics of Alcoholism. Microsatellite data from 143 families ascertained through having three or more individuals affected with alcohol dependence were used for this investigation. Four regions showing evidence for linkage were identified using single-locus models from previous investigations. We investigated the genetic linkage, pattern of disease inheritance, and pair-wise genetic epistasis of these loci using the TLINKAGE program for two-disease-locus analysis.

## Background

Many human diseases, such as cancers and psychiatric disorders, are caused by more than one gene. Analysis using two-locus methods is a logical method of adequately modeling genetically complex traits that are caused by two or more genes. Schork et al. compared the parametric one-locus and two-locus LOD-score analyses and found that linkage analysis with two-locus methods had higher power to detect linkage than did those with one-locus models for a trait governed by two genes [1]. However, other researchers argue that single-locus models are more advantageous than two-locus models in such situations [2-4]. In addition to power issue, two-locus methods more accurately estimate trait-locus positions than do single-locus methods and may be able to detect possible genetic epistasis between loci, which can lead to a better understanding of patterns of disease inheritance. Furthermore, Frankel and Sckork pointed out that there exist two-locus models for which genes are not detectable using any single-locus model, and only two-locus models could reveal genetic components in these cases [5]. There are two recent comprehensive publications about two-disease-locus models: Strauch et al., who suggest a procedure to maximize the power to detect linkage for complex traits using two-locus methods [6], and Li and Reich, who systematically compared 512 two-locus, two-allele disease models [7].

Using segregation analysis, Yuan et al. suggested a multi-locus etiology for alcoholism [8]. Furthermore, Begleiter et al. proposed that alcohol dependence is likely influenced by a variety of genetic and environmental risk factors, such as a reduced P300 amplitude and tobacco addiction [9]. P300 is a positive brain wave that occurs about 300 milliseconds after a stimulus. LOD scores, assuming heterogeneity (HLOD), were calculated on the basis of evidence. A genome-wide scan of the data from the Collaborative Study on the Genetics of Alcoholism (COGA) was conducted to detect genetic linkage to the chromosomal regions responsible for alcoholism susceptibility in the Genetic Analysis Workshop 11.

In this work, we identified four regions on different chromosomes showing evidence for linkage using single-locus methods as described in the literature [10]. On the basis of these markers, we conducted an exploratory study of investigating the possible disease inheritance patterns and further evidence of genetic linkage using model-based two-locus methods. We further analyzed the evidence of genetic interaction between two disease loci by comparing the LOD scores using two-locus models and single-locus models on the two disease loci.

## Methods

### Data

We analyzed COGA microsatellite data for a 10-cM genome scan. The family data of COGA included 1,614 individuals of 143 pedigrees ascertained for having three or more family members affected with alcohol dependence. COGA criteria were used for diagnosis throughout the study for ascertainment. Marker allele frequencies were estimated from the data. We identified four markers showing evidence of linkage using single-locus methods as described in the literature [10] and included them in the two disease-locus linkage analysis. The markers were D1S1595, D6S1006, D7S1797, and D15S642 on chromosomes 1, 6, 7, and 15, respectively.

### Parametric analysis using two-locus methods

The TLINKAGE program was used for two-disease-locus analyses in this report [1]. TLINKAGE is an extension of LINKAGE based on the Elston-Stewart algorithm [11] and can accommodate large pedigrees with two multi-allelic markers in the analysis. The program assumes both trait loci are diallelic and on different chromosomes. For two-disease-locus/one-marker-locus analyses, we assumed that one disease locus was linked to a marker at recombination rate θ and the other disease locus was not linked to the tested marker locus. The LOD score was computed as follows:

LOD(θ) = log_10 _{ L(θ, 0.5) / L(0.5, 0.5) } ; 0 ≤ θ ≤ 0.5.

The product of 2 ln(10) and the maximized LOD score is an asymptotically 50:50 mixture of a point probability mass at 0 and a chi-squared distribution with one degree of freedom under the null hypothesis of θ = 0.5.

A loss of power due to incorrect specification of models and misspecification of disease transmission inheritance is always a concern for parametric approaches to linkage analysis. Despite its power loss, Risch et al. [12] and Risch and Giuffra [13] suggested that parametric linkage analysis is robust to misspecification of genetic models, provided that the dominance is correctly specified.

In this study, we considered five types of two-locus models: dominant/dominant, dominant/recessive, recessive/dominant, recessive/recessive, and modified dominant/dominant, in which the penetrance of double heterozygotes is half that of genotypes having three or four disease alleles. We assumed that two disease loci had the same effects on the trait; thus, the penetrance matrix was symmetric. The phenocopy rate was fixed at 0.05. A wide range of penetrance values and allele frequencies on the disease loci were carried out in the TLINKAGE program for each of the five models.

Heterogeneity was considered a possible confounder for alcoholism [10]. The simplest approach to analyzing suspected genetic heterogeneity is to partition the families into subgroups in which simpler genetic models might suffice. Here, heterogeneity was tested using program HOMOG [14]. We calculated the HLOD score on the basis of the evidence of genetic heterogeneity for each of the four markers. The evidence of genetic epistasis was analyzed by comparing the LOD score using two-locus models and the LOD scores using single-locus models on the two disease loci.

## Results

We first performed TLINKAGE for two-disease-locus/one-marker-locus analysis for each of the four markers D1S1595, D6S1006, D7S1797, and D15S642. Markers D1S1595 and D6S1006 were selected on the basis of the high LOD score, D7S1797 on the basis of its z-score, and D15S642 on the basis of its nonparametric linkage score. No LOD scores for D1S1595, D6S1006, or D7S1797 were higher than 0.2 for any of the five models. All the LOD scores for these markers were close to 0 for the recessive/recessive models. D15S642 for the dominant/dominant models showed the strongest evidence of linkage among the four markers and five genetic models. Some of the LOD scores were 2.0 or more.

It is noteworthy that, for D7S1797 and D15S642, some families had strong evidence of linkage, whereas others had negative LOD scores in the dominant/dominant models, which resulted in the smaller overall LOD scores. These phenomena suggest genetic heterogeneity. Therefore, we tested heterogeneity using the program HOMOG for these two markers. For D7S1797, the HLOD score was 1.8 with 15 linked families (10%). The HLOD score with 31 linked pedigrees (20%) was 4.47 for D15S642.

We then calculated the corresponding HLOD scores using single-locus models. The HLOD scores were 2.0 and 4.29 for D7S1797 and D15S642, respectively. There were no significant discrepancies between the HLOD scores for D7S1797 and D15S642 using single-locus models and two-disease-locus models. We believe that neither D7S1797 nor D15S642 interacted with other postulated disease loci. A broad range of penetrance values and disease allele frequencies were carried out using TLINKAGE. In Table 1, we present the results of the highest HLOD scores for D7S1797 and D15S642.

## Discussion

Results from our two-disease-locus model analysis using TLINKAGE did not support the evidence of linkage to the disease susceptibility loci on chromosomes 1 and 6 that had been previously identified using a single-locus method. This may be due to the program employed (GENEHUNTER versus TLINKAGE) or that there are more than two disease loci for alcohol dependence (neither single-locus models nor two-locus models are robust enough to detect linkage). Lin et al. suggested two-locus models might be too simple to analyze the underlying true model [15].

The patterns of LOD scores by pedigrees for the markers D7S1797 and D15S642 revealed the evidence of genetic heterogeneity for the dominant/dominant models. Assuming heterogeneity, our findings suggest evidence of linkage to the disease susceptibility loci on chromosomes 7 and 15. There are no gene × gene interactions between D7S1797 and D15S642 and other postulated disease loci on the basis of our findings.

The results may suggest that the underlying true model for alcoholism is more complicated than the two-locus models are able to explain. More general multilocus methods may be required to adequately model the effects of major genes, polygenes, and environmental factors for alcohol dependence.

## Conclusion

Two-locus analysis is complex because of the specification of several parameters. However, it may not be powerful for traits with very complex genetic etiology. Previous studies [12,13] suggested that a power loss of parametric approaches could be reduced provided that dominance is correctly specified. On the basis of this, we proposed five types of two-locus models, four of which were involved with dominance type and allowed a broad range of penetrance values and allele frequencies carried out using TLINKAGE. The signals obtained using recessive/recessive models were generally weaker than were those obtained by the other four models in our analysis. Therefore, we recommend reducing the possibility of a gross power loss due to misspecification of genetic models by using this approach.

## Abbreviations

COGA: Collaborative Study on the Genetics of Alcoholism

HLOD: Heterogenecity LOD

## Authors' contributions

Both authors contributed equally to this work.

## Figures and Tables

**Table 1:** Summary of HLOD scores for markers D7S1797 and D15S642

Marker	θ	Single-locus HLOD	Two-locus HLOD	Proportion of linked pedigrees
D15S642	0.0	4.29	4.47	20%
D7S1797	0.0	2.0	1.8	10%
